# Dopamine D1/D2 Receptor Activity in the Nucleus Accumbens Core But Not in the Nucleus Accumbens Shell and Orbitofrontal Cortex Modulates Risk-Based Decision Making

**DOI:** 10.1093/ijnp/pyv043

**Published:** 2015-04-23

**Authors:** Bettina Mai, Susanne Sommer, Wolfgang Hauber

**Affiliations:** Department Animal Physiology, University of Stuttgart, Stuttgart, Germany (Mrs Mai, Sommer, and Dr Hauber).

**Keywords:** Decision making, dopamine, nucleus accumbens, orbitofrontal cortex, rat, risk

## Abstract

**Background:**

It is well known that brain dopamine (DA) signals support risk-based decision making; however, the specific terminal regions of midbrain DA neurons through which DA signals mediate risk-based decision making are unknown.

**Methods:**

Using microinfusions of the D1/D2 receptor antagonist flupenthixol, we sought to explore the role of D1/D2 receptor activity in the rat orbitofrontal cortex (OFC) and core and shell regions of the nucleus accumbens (AcbC and AcbS, respectively) in the regulation of risky choices. A risk-discounting task was used that involves choices between a certain small-reward lever that always delivered 1 pellet or a risky large-reward lever which delivered 4 pellets but had a decreasing probability of receiving the reward across 4 subsequent within-session trial blocks (100%, 50%, 25%, 12.5%). To validate task sensitivity to experimental manipulations of DA activity, we also examined the effects of systemic amphetamine and flupenthixol.

**Results:**

Systemic amphetamine increased while systemic flupenthixol reduced risky choices. Results further demonstrate that rats that received intra-AcbC flupenthixol were able to track increasing risk associated with the risky lever but displayed a generally reduced preference for the risky lever across all trial blocks, including in the initial trial block (large reward at 100%). Microinfusions of flupenthixol into the AcbS or OFC did not alter risk-based decision making.

**Conclusions:**

Our data suggest that intra-AcbC D1/D2 receptor signaling does not support the ability to track shifts in reward probabilities but does bias risk-based decision making. That is, it increased the rats’ preference for the response option known to be associated with higher risk-related costs.

## Introduction

For adaptive decision making in an uncertain and ever-changing environment, animals often have to analyze expected costs and benefits of the available response options, as for instance the probability and magnitude of rewards associated with alternative courses of action. The capacity to assess the probability (risk) of receiving rewards relies on a complex neural network including the medial prefrontal cortex (mPFC), orbitofrontal cortex (OFC), core and shell regions of the nucleus accumbens (AcbC and AcbS, respectively), and basolateral amygdala (BLA; Cardinal, 2006; Roitman and Roitman, 2010; Stopper and Floresco, 2011; Zeeb and Winstanley, 2011; Abela and Chudasama, 2013; Morgado et al., 2014). For instance, electrophysiological studies revealed neurons within the OFC that code information about reward risk, defined as variance in the spread of outcomes (O’Neill and Schultz, 2010). Moreover, inactivation of the mPFC, the AcbC/AcbS, or BLA altered the preference for the risky reward relative to the certain reward in probabilistic tasks in which risk refers to choice situations with known distributions of potential reward related to particular actions (Cardinal and Howes, 2005; Ghods-Sharifi et al., 2009; St Onge and Floresco, 2010; Stopper and Floresco, 2011). A considerable body of evidence suggests that the mesocorticolimbic dopamine (DA) system supports risk-based decision making. For instance, midbrain DA neurons are activated by stimuli predicting risky rewards and generate a neuronal signal that varies monotonically with risk (Fiorillo et al., 2003). Such DA risk signals may have several functions: they could provide input to brain structures involved in the evaluation of reward and risk and serve as teaching signals to enhance learning (Schultz, 2010). Furthermore, there is evidence that DA receptor expression patterns can predict risk bias: lower striatal D2 receptor mRNA expression in a subgroup of adolescent rats with greater risk-taking correlates with greater cocaine self-administration in adulthood (Mitchell et al., 2014). Moreover, drug-induced manipulations of DA activity affect risk-based decision making. For instance, sustained alcohol intake can alter risk-based decision making in rats by compromising DA signaling of risk (Nasrallah et al., 2011).

Terminal regions of midbrain DA neurons, through which DA signals mediate risk-based decision making, have been identified in part. For instance, mPFC DA receptors contribute in a dissociable manner to risk-based decision making: an intra-mPFC D1-receptor blockade reduced risky choices whereas an intra-mPFC D2-receptor blockade increased them (St Onge et al., 2011). Moreover, a previous receptor type–specific analysis revealed that a D1, but not D2, receptor blockade within the whole Acb reduced risky choices (Stopper et al., 2013).

The role of DA signals in the AcbC, AcbS, and OFC in risk-based decision making remains to be addressed. Therefore, by means of a probabilistic choice task used in previous work (St Onge et al., 2010), the present study sought to explore whether D1/D2 receptor activity in the AcbC, AcbS, or OFC supports risk-based decision making using local microinfusions of flupenthixol. To validate task sensitivity to experimental manipulations of DA activity, we also examined the effects of systemic amphetamine and flupenthixol, two prototypical DAergic drugs with prominent effects on risky choice.

## Methods

All animal experiments were performed according to the German Law on Animal Protection and approved by the proper authorities.

### Experiment 1: Effects of Flupenthixol and Amphetamine on Risky Choice

#### Subjects

Male Lister hooded rats (Charles River) weighting between 200 and 225g upon arrival were used. They were housed in transparent plastic cages (60cm × 38cm × 20cm, Tecniplast) in groups of up to four animals. Rats had a 12:12-h light-dark cycle (lights on at 07:00) with *ad libitum* access to water. Upon arrival, standard laboratory chow (Altromin) was given *ad libitum* for at least 5 days. To maintain rats at approximately 85% of their free-feeding weight, food was restricted to 15g per animal per day. For environmental enrichment, a plastic tube (20cm, Ø 12cm) was fixed on the lid of each cage. Temperature (22±2°C) and humidity (50±10%) were kept constant in the animal house.

#### Apparatus, Habituation, and Lever Press Training

Training and testing took place in identical operant chambers (24 x 21 x 30cm; Med Associates), which were surrounded by sound attenuating cubicles (for details see Supplementary Material). Habituation and lever press training was as described in Mai and Hauber (2012; see also Supplementary Material). After termination of lever press training, the risk-discounting task was introduced.

#### Risk Discounting Task

**Figure 1.**
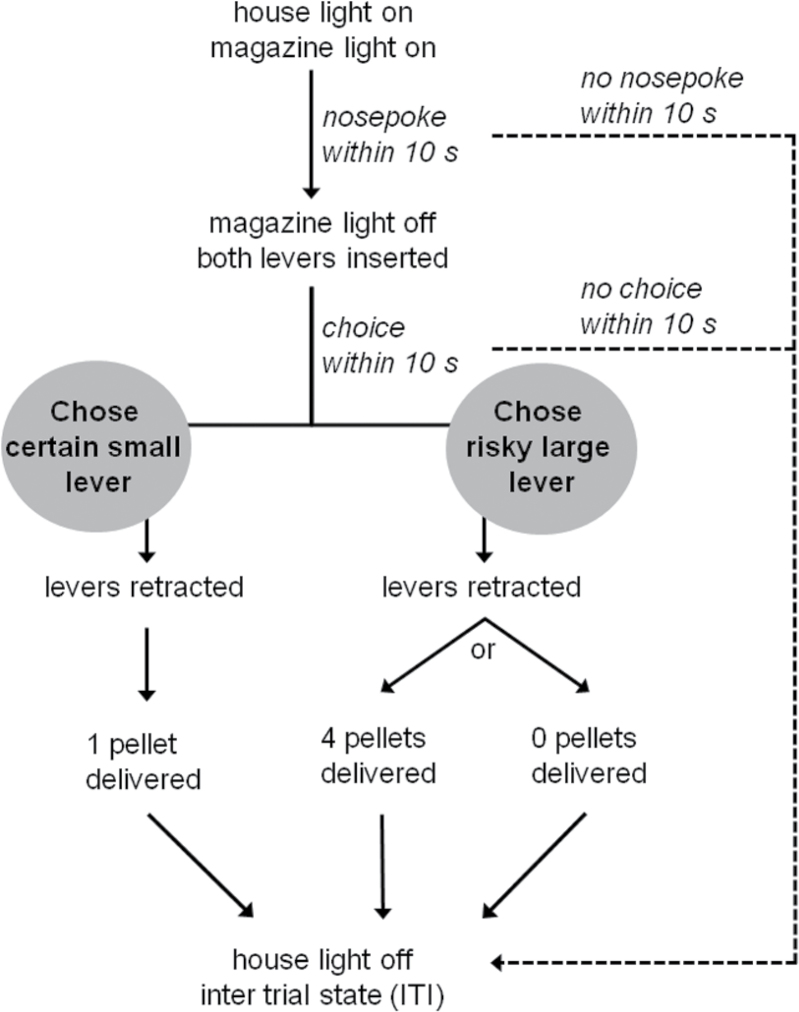
Schematic of a single free-choice trial in the risk-discounting task. Choice of the certain lever always led to delivery of one pellet, while choice of the risky lever led to delivery of 4 pellets with decreasing probabilities across trial blocks (*p* = 1.0, 0.5, 0.25, 0.125).

The risk-discounting task used here was based on protocols described by Cardinal and Howes (2005) and St Onge and Floresco (2009). For each animal, one of the two levers was designated as being the certain small-reward lever and the other therisky large-reward lever. This assignment remained constant for each animal throughout the study and was counterbalanced across rats. Choice of the certain lever always delivered one pellet and choice of the risky lever had a probabilistic delivery of four or no pellets. The probabilistic delivery of the risky lever changed in the course of the daily sessions. The task is described in detail in the Supplementary Material. A schematic of a single free-choice trial in the risk-discounting task is given in Figure 1.

#### Effects of D-Amphetamine and Flupenthixol: Behavioral Procedures

Animals were trained on the risk-discounting task for 15 days before the effects of d-amphetamine and flupenthixol on risk discounting were assessed. Different doses of d-amphetamine hemisulfate salt (Sigma Aldrich) and cis-(Z)-Flupenthixol dihydrochloride (Sigma Aldrich) were investigated in two subsequent experiments. In the first experiment, higher drug doses (amphetamine 2mg/kg, flupenthixol 0.4mg/kg vs. saline 1ml/kg) were tested; in the second experiment, lower drug doses (amphetamine 0.5mg/kg, flupenthixol 0.25mg/kg vs. saline 1ml/kg) were tested. Drugs were dissolved in physiological saline and administered intraperitoneally at a volume of 1ml/kg. After administration rats were returned to their home cages for 30min (amphetamine), 45min (saline), or 60min (flupenthixol) until behavioral testing. A within-subject design was used in each experiment, with the order of drug administration pseudo-randomized across rats. Drugs (saline, amphetamine, flupenthixol) in each experiment were tested in two subsequent 4-day blocks (see Supplementary Material).

#### Data Analysis and Statistics

Percentage choices of the risky reward lever are given as means ± standard error of the mean (SEM). Data were subjected to a repeated-measures analysis of variance (ANOVA) with two within-subject factors (treatment and large reward probability). As a within-subject protocol was used for each experiment, all rats received all treatments in a randomly varied order. All statistical computations were carried out with STATISTICA TM (version 7.1, StatSoft, Inc.). The level of statistical significance (α-level) was set at *p* ≤ 0.05 (α-levels > 0.05 were designated as n.s., not significant).

### Experiment 2: Effects of AcbC Flupenthixol on Risky Choice

In Experiment 2, animals were trained on the risk-discounting task until they reached the defined learning criterion as described in Experiment 1. Thereafter, animals were implanted with cannulae directed to the AcbC using standard stereotaxic procedures (see Supplementary Material). The following coordinates were chosen with reference to the atlas of Paxinos and Watson (1998): anterioposterior (AP) +1.2mm; mediolateral (ML) ±1.8mm; dorsoventral (DV) -7.0mm. After recovery, effects of flupenthixol microinfusions on risky choices were assessed. We conducted further analyses of behavioral data to obtain more detailed information as to how this treatment could have induced changes in risky choices (see Supplementary Material).

#### Microinfusion Procedure

Two doses of flupenthixol (15; 17.5 µg) were examined in two subsequent tests. A within-subject design was used where the order of drug (cis-(Z)-flupenthixol dihydrochloride; Sigma Aldrich) and saline microinfusion was counterbalanced for each rat. The sample size in the first test (flupenthixol 15 µg) was n = 13 and in the second test (flupenthixol 17.5 µg) n = 10, because three rats were excluded due to occluded guide cannulae. Microinfusions (procedure, see Supplementary Material) were separated by risk-discounting task-training days without microinfusions. Doses refer to previous studies demonstrating behavioral effectiveness after intra-AcbC infusion (Moscarello et al., 2010; Saunders and Robinson, 2012).

#### Histology

After completion of the behavioral testing, animals were euthanized by an overdose of isoflurane (Abbott) to control for correct cannula placements. Brains were removed, fixed in 4% formalin for 24h, and stored in 30% glucose. Brains were frozen and coronal brain sections (35–40 µm) were collected, mounted on coated slides, and stained with cresyl violet. Placements of guide cannulas were verified with reference to the atlas of Paxinos and Watson (1998).

### Experiment 3: Effects of AcbS Flupenthixol on Risky Choice

Unless noted otherwise, the same procedures as described in Experiment 2 were used. Animals were implanted with cannulae directed to the AcbS (AP +1.2mm; ML ±0.9mm; DV -7.0mm; Paxinos and Watson, 1998) and, after recovery, the effects of flupenthixol (17.5 µg) microinfusions on risky choices were assessed.

### Experiment 4: Effects of OFC Flupenthixol on Risky Choice

Unless noted otherwise noted, the same procedures as in Experiments 2 and 3 were used. After completion of lever pressing training, animals were subsequently trained in the risk-discounting task. Animals were implanted with cannulae directed to the OFC (AP +3.5mm; ML ±2.6mm; DV -5.2; Paxinos and Watson, 1998).

## Results

### Experiment 1: Effects of Flupenthixol and Amphetamine on Risky Choice

**Figure 2.**
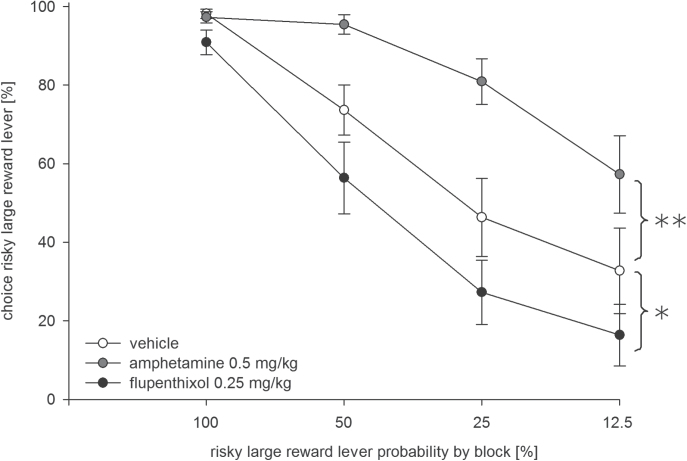
Effects of amphetamine (grey symbols, 0.5mg/kg, i.p.) and flupenthixol (black symbols, 0.25mg/kg, i.p.) on probabilistic choice. Mean (± standard error of the mean) percentages of risky lever choices per session are shown across decreasing probabilities to obtain the risky reward within one session (n = 11). *p < 0.05; **p < 0.001 (significant main effects of treatment).

In Experiment 1, the effects of flupenthixol and amphetamine were tested against saline (n = 11). Results demonstrated an increased preference for the risky lever in amphetamine-treated rats (0.5mg/kg) and a reduced preference in flupenthixol-treated rats (0.25mg/kg; Figure 2). Accordingly, an ANOVA revealed a main effect of treatment [F(2, 20) = 26.43, *p* < 0.001] and trial block [F(3, 30) = 51.83, *p* < 0.001] and a treatment x block interaction [F(6, 60) = 5.16, *p* < 0.001]. Further analysis of data from amphetamine- and saline-treated animals revealed significant effects of treatment [F(1, 10) = 26.07, *p* < 0.001] and trial block [F(3, 30) = 28.17, *p* < 0.001] and a treatment x block interaction [F(3, 30) = 6.35, *p* < 0.01]. Inspection of data from flupenthixol- and saline-treated animals showed significant effects of treatment [F(1, 10) = 6.53, *p* < 0.05] and trial blocks [F(3, 30) = 53.58, *p* < 0.001], but no treatment x block interaction [F(3, 30) = 0.65, n.s.].

Animals treated with flupenthixol and amphetamine showed increased response latencies. An ANOVA revealed a main effect of treatment [F(2, 20) = 7.87, *p* < 0.01] and trial block [F(3, 30) = 6.06, *p* < 0.01] and a treatment x block interaction [F(6, 60) = 9.91, *p* < 0.001]. An analysis of latency data from amphetamine- and saline-treated animals showed no treatment effect [F(1, 10) = 0.004, n.s.], a main effect of block [F(3, 30) = 4.41, *p* < 0.05], and a trend for a treatment x block interaction [F(3, 30) = 2.33, *p* = 0.09]. An inspection of data from flupenthixol- and saline-treated animals revealed a treatment effect [F(1, 10) = 20.74, *p* < 0.01], a main effect of block [F(3, 30) = 10.50, *p* < 0.001] and a treatment x block interaction [F(3, 30) = 8.36, *p* < 0.001].

### Experiment 2: Effects of AcbC Flupenthixol on Risky Choice

**Figure 3.**
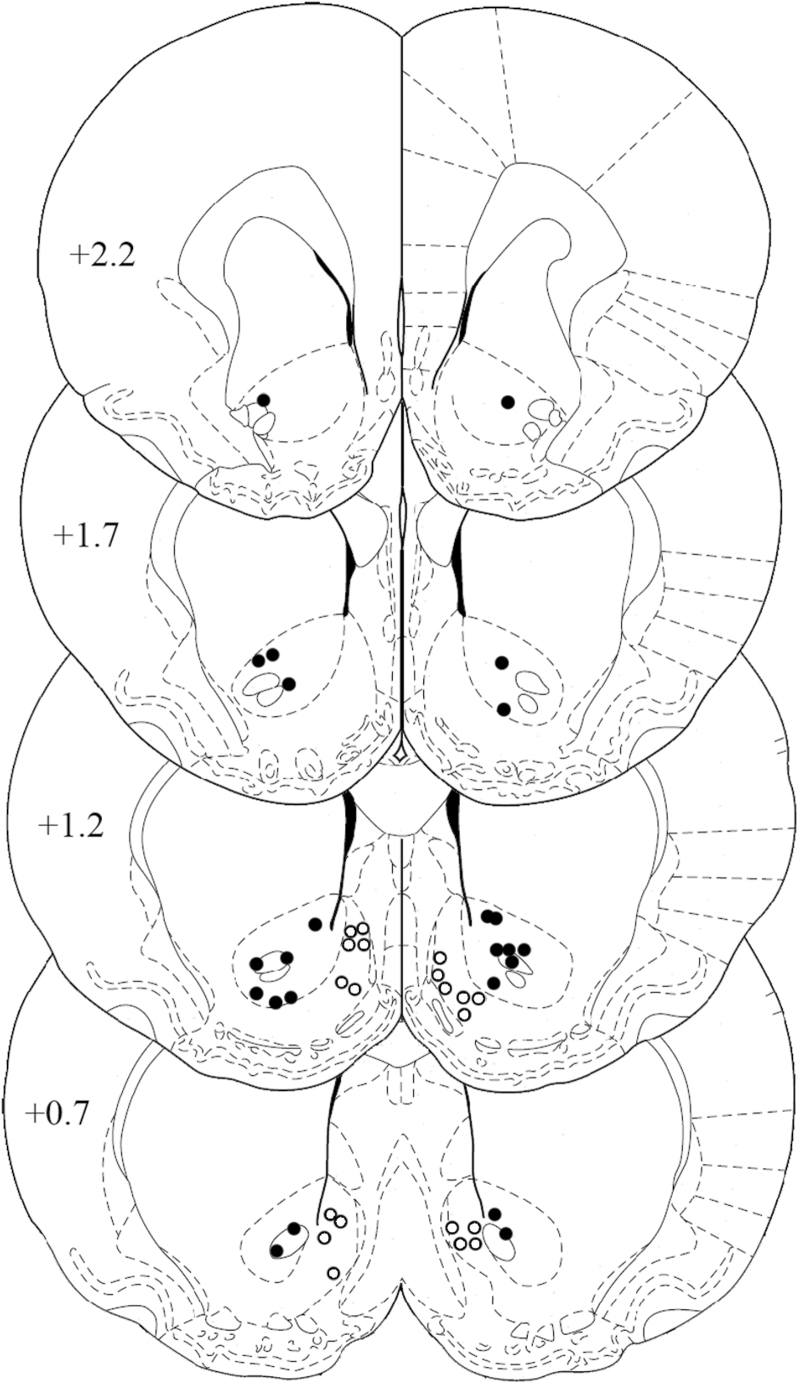
Cannulae placement in the nucleus accumbens. The schematic shows the location of microinfusion cannulae tips in the nucleus accumbens core (filled circles) and shell (open circles). The numbers indicate the distances from bregma, in millimeters.

Animals were trained on the risk-discounting task for 26 days before being implanted with guide cannulae. The location of infusion cannulae tips from all rats of Experiment 2 is shown in Figure 3; no animals were excluded because of misplaced cannulae. Intra-AcbC flupenthixol (15 µg, n = 13) did not alter risk-based decisions (data not shown). In line with this notion, an ANOVA revealed no main effect of treatment [F(1, 12) = 0.76, n.s.], a main effect of trial block [F(3, 36) = 35.92, *p* < 0.001], and no treatment x block interaction [F(3, 36) = 0.17, n.s.]. In addition, an analysis of latency data showed no main effect of treatment [F(1, 12) = 0.10, n.s.], a main effect of block [F(3, 36) = 4.14, *p* < 0.05], and no treatment x block interaction [F(3, 36) = 0.59, n.s.].

**Figure 4.**
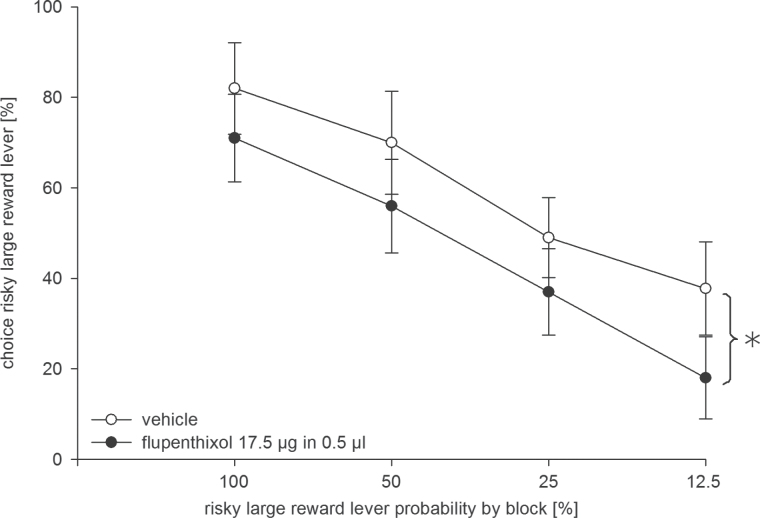
Effects of dopamine receptor blockade in the nucleus accumbens core (AcbC) on probabilistic choice in rats. Mean (± standard error of the mean) percentages of risky lever choices per session are shown for decreasing probabilities to obtain the risky reward within one session following intra-AcbC infusion of flupenthixol (17.5 µg/0.5 µl, black circles) or vehicle (0.5 µl, white circles; n = 10). *p < 0.05 (significant main effect of treatment).

By contrast, intra-AcbC flupenthixol (17.5 µg, n = 10) reduced preference for the risky lever (Figure 4). Accordingly, an ANOVA revealed a main effect of treatment [F(1, 9) = 5.97, *p* < 0.05] and trial block [F(3, 27) = 25.69, *p* < 0.001], but no treatment x block interaction [F(3, 27) = 0.18, n.s.]. Intra-AcbC flupenthixol increased response latencies. An ANOVA revealed no main effect of treatment [F(1, 9) = 0.66, n.s.], but a significant trial block [F(3, 27) = 5.09, *p* < 0.01] and treatment x block interaction [F(3, 27) = 3.10, *p* < 0.05].

For a more detailed characterization of treatment effects, we also compared trial omissions and session durations after intra-AcbC flupenthixol vs. saline infusion. ANOVA revealed no treatment effects on trial omissions [vehicle, 5.7±4.4; flupenthixol, 4.3±2.2; F(1, 9) = 0.08, n.s.] and session duration [vehicle, 56.1±3.7min; flupenthixol, 55.1±2.2; F(1, 9) = 0.06, n.s.]. Regarding the win-stay ratios, an ANOVA revealed an almost significant effect of treatment [vehicle, 0.81±0.05; flupenthixol, 0.62±0.11; F(1, 8) = 5.27, *p* = 0.05], indicating a reduced reward sensitivity in flupenthixol-treated rats. By contrast, an analysis of lose-shift ratios revealed no treatment effect [vehicle, 0.46±0.11; flupenthixol, 0.56±0.09; F(1, 9) = 0.99, n.s].

In a control experiment in experimentally naïve rats, we examined the effects of intra-AcbC flupenthixol (17.5 µg) on reward magnitude discrimination using a task variant in which subjects could choose between two levers that delivered either 4 pellets with a decreasing probability of reward or 1 pellet with a certain reward (see Supplementary Material). Results showed that intra-AcbC flupenthixol (17.5 µg) did not affect reward magnitude discrimination (Supplementary Figure S1).

### Experiment 3: Effects of AcbS Flupenthixol on Risky Choice

**Figure 5.**
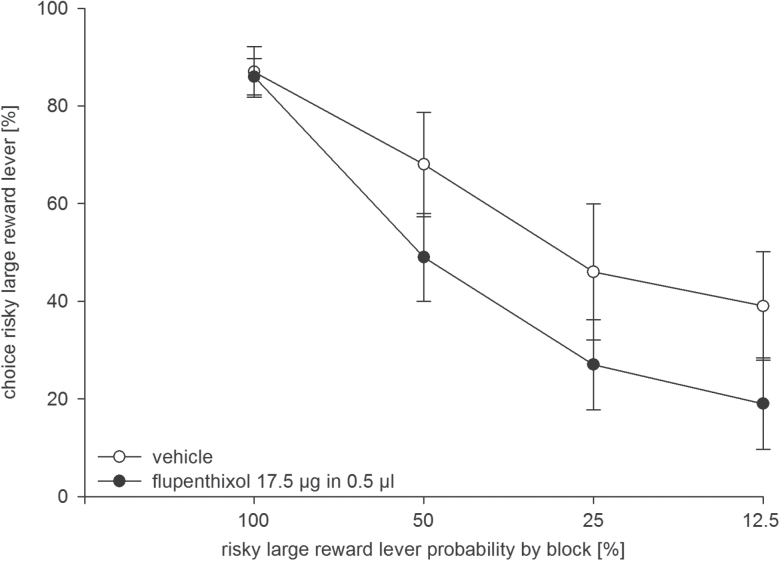
Effects of dopamine receptor blockade in the nucleus accumbens shell (AcbS) on probabilistic choice. Mean (± standard error of the mean) percentages of risky lever choices per session are shown for decreasing probabilities to obtain the risky reward within one session following infusion of flupenthixol (17.5 µg/0.5 µl, black circles,) or vehicle (0.5 µl, white circles; n = 10).

Animals were trained on the risk-discounting task for 22 days before being implanted with guide cannulae. The location of infusion cannulae tips from all animals of Experiment 3 (n = 10) is shown in Figure 3. One animal was excluded due to cannulae misplacement, and another one failed to display risk discounting and was also excluded from analyses. Results show that intra-AcbS flupenthixol (17.5 µg) did not alter the preference for the risky lever (Figure 5). An ANOVA revealed no main effect of treatment [F(1, 9) = 3.13, *p* = 0.11], but a significant effect of trial block [F(3, 27) = 28.39, *p* < 0.001], and no treatment x block interaction [F(3, 27) = 1.35, *p* = 0.3]. Of note, in these rats there was a subgroup (n = 4 out of 10) that, under control conditions, had a preference for the risky lever (≥50%) in the 12.5% trial block. In this subgroup, intra-AcbS flupenthixol reduced the preference for the risky lever. An ANOVA revealed an almost significant effect of treatment [F(1, 3) = 8.66, *p* = 0.06], a main effect of trial block [F(3, 9) = 5.32, *p* < 0.05], and a treatment x block interaction [F(3, 9) = 5.14, *p* < 0.05].

Intra-AcbS flupenthixol did not affect response latencies, trial omissions, or win stay/lose shift tendencies (see Supplementary Material).

### Experiment 4: Effects of OFC Flupenthixol on Risky Choice

**Figure 6.**
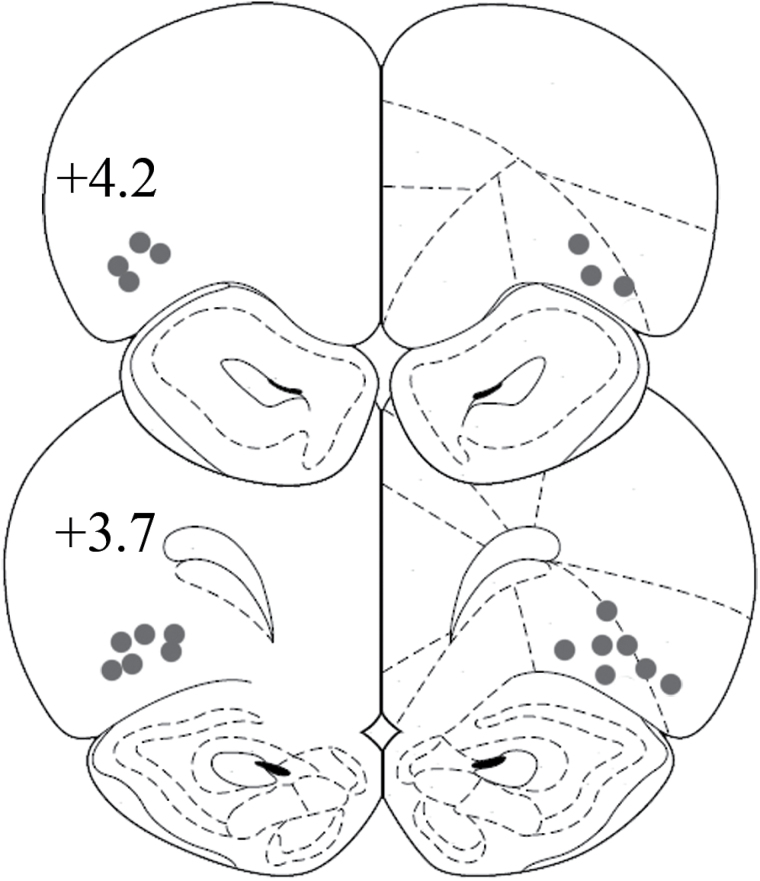
Cannulae placement in the orbitofrontal cortex. The schematic shows the location of microinfusion cannulae tips. The numbers indicate the distances from bregma, in millimeters.

**Figure 7.**
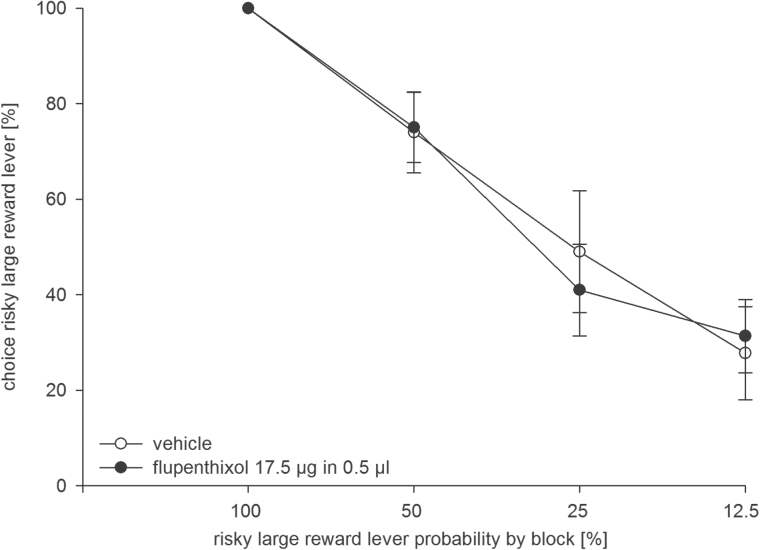
Effects of dopamine receptor blockade in the orbitofrontal cortex on probabilistic choice. Mean (± standard error of the mean) percentages of risky lever choices per session are shown for decreasing probabilities to obtain the risky reward within one session following infusion of flupenthixol (17.5 µg/0.5 µl, black circles) or vehicle (0.5 µl, white circles; n = 10).

Animals were trained on the risk-discounting task for 25 days before being implanted with guide cannulae. Placements of infusion cannulae tips from all animals (n = 10) of Experiment 4 are shown in Figure 6. As shown in Figure 7, intra-OFC infusion of flupenthixol (17.5 µg) did not alter the preference for the risky lever. Accordingly, an ANOVA revealed no main effect of treatment [F(1, 9) = 0.03, n.s.], but a significant effect of trial block [F(3, 27) = 36.88, *p* < 0.001], and no treatment x block interaction [F(3, 27) = 0.28, n.s.]. Intra-OFC flupenthixol did not affect response latencies. An ANOVA revealed no main effect of treatment [F(1, 9) = 1.29, n.s.], but a significant effect of trial block [F(3, 27) = 7.69, *p* < 0.001], and no treatment x block interaction [F(3, 27) = 0.29, n.s.].

## Discussion

The main finding of this study is that in rats tested in a probabilistic choice task, the preference for a risky reward was reduced after an intra-AcbC D1/D2 receptor blockade, suggesting that DA activity in the AcbC plays a key role in promoting choices of risky reward.

### Systemic Dopamine Manipulation and Risky Choice

In line with previous reports (e.g. Cardinal and Howes, 2005; St Onge and Floresco, 2009), control animals in all experiments showed risk discounting across within-session trial blocks with decreasing large-reward probabilities. In particular, as predicted by the value-matching law (Herrnstein, 1961), they displayed rational choices: they showed no preference for either the risky or certain lever in the neutral trial block (4 pellets at *p* = 0.25 vs. 1 pellet at *p* = 1.0) but avoided the risky lever in the trial block where the large reward was most uncertain (*p* = 0.125).

To validate task sensitivity to experimental manipulations of DA activity, we examined the effects of systemic amphetamine and flupenthixol, two prototypical DAergic drugs with prominent effects on risky choices. In line with a previous work (St Onge and Floresco, 2009), amphetamine (0.5mg/kg) increased the preference for the risky lever when the odds of obtaining a larger reward decreased over a session. Conversely, amphetamine decreased the preference for the risky lever when the odds of obtaining a larger reward increased over a session, suggesting that amphetamine may impair the ability to shift preference away from or towards risky options upon changes in probabilistic reward value (St Onge et al., 2010). By contrast, flupenthixol (0.25mg/kg) reduced the preference for the risky lever and increased response latencies. Overall, our data are consistent with previous work and confirm task sensitivity to changes in DA activity.

### Nucleus Accumbens Dopamine and Risky Choice

Flupenthixol (17.5 µg) administered to the AcbC significantly reduced the preference for the risky lever in Experiment 1, suggesting that drug actions in the AcbC play a role in mediating behavioral effects of systemic flupenthixol. Drug actions into adjacent subregions, due to diffusion into the AcbS, could contribute to the observed effects. However, because intra-AcbC and AcbS infusions of flupenthixol in comparable doses and volumes produced dissociable behavioral effects, drug spread across Acb subregions may be limited (Ito and Hayen, 2011).

Remarkably, microinfusion of flupenthixol into the AcbC reduced the preference for the risky lever across all trial blocks, including the initial trial block with the large reward at *p* = 1.0, a finding that points to a drug-induced general increase of risk aversion. Importantly, our results indicate that flupenthixol-induced impairments of reward magnitude discrimination could not account for these findings. Accordingly, a large number of studies revealed that transient or permanent inactivation of the AcbC did not alter (e.g. Balleine and Killcross, 1994; Giertler et al., 2003, 2004; Cardinal and Howes, 2005) or moderately enhanced (Cardinal and Cheung, 2005) reward magnitude discrimination. Similarly, systemic DA antagonists do not affect the perceived quantity of food (Martin-Iverson et al., 1987). Likewise, Acb DA depletion (Salamone et al., 1994, 2001; Cousins et al., 1996; Mai and Hauber, 2012) or intra-Acb microinfusion of DA antagonists (Hauber et al., 2000; Calaminus and Hauber, 2007; Stopper et al., 2013) did not affect the ability to discriminate large from small rewards. Consistent with these findings, optogenetic stimulation of Acb DA signaling did not influence reward magnitude–based decisions (Saddoris et al., 2014).

While our subregion-specific analysis demonstrates that a blockade of D1/D2 receptors in the AcbC reduced risky choices, a previous receptor type–specific analysis revealed that a D1, but not D2, receptor blockade within the whole Acb reduced risky choices (Stopper et al., 2013). Together, these findings suggest that AcbC D1 receptors may play a key role in regulating risk-based decision making. Furthermore, intra-AcbC flupenthixol (17.5 µg) increased response latencies, a finding that is consistent with the notion that the Acb and its DA input invigorate instrumental responding (e.g. Yun et al., 2004; Salamone et al., 2005; Calaminus and Hauber, 2007).

In line with earlier data (e.g. Stopper et al., 2013), under vehicle treatment our rats chose the risky lever in about 85% of trials after receiving the risky reward in the preceding trial, while they shifted to the certain lever in about 35–50% of trials after not receiving the risky reward in the preceding trial. Intra-AcbC flupenthixol selectively decreased win-stay tendencies but left lose-shift tendencies unaltered. According to this analysis, reduced risky choice under flupenthixol is predominantly driven by an underestimated probability for a large reward re-gain in the subsequent trial. However, our interpretation is limited because this win-stay/lose-shift analysis relies on the arguable assumption that a given choice trial was influenced only by the immediately preceding choice trial and, due to data limitations, does not allow a separate analysis of individual within-session trial blocks. Contrasting with our results, reduced risky choice after SCH23390 injection into the whole Acb was associated with unaltered win-stay but enhanced lose-shift tendencies (Stopper et al., 2013). Of note, transient Acb inactivation (Stopper and Floresco, 2011) and Acb D1 receptor blockade (Stopper et al., 2013) both reduced risky choice associated with either reduced win-stay or increased lose-shift tendencies. Thus, reduced risk taking after experimental Acb manipulations may not necessarily align with uniform changes in win-stay/lose-shift tendencies. Overall, our data suggest that intra-AcbC flupenthixol reduced risky choice and win-stay tendencies.

Results further show that intra-AcbS flupenthixol did not alter risk-based decision making. It is unlikely that drug dosing (17.5 µg/side) was inappropriate, as this dose was behaviorally effective when injected into the AcbC (Experiment 2) and this or a moderately lower dose was behaviorally effective when administered to the AcbC (Naneix et al., 2009; Simmons and Neill, 2009; Moscarello et al., 2010). Nevertheless, we cannot exclude the possibility that higher doses could impair risky choice. Remarkably, in drug-naïve rats’ D1 mRNA expression, but not D2 mRNA expression, in the AcbS was positively correlated with risk-taking in a task that involves a certain small reward and a large reward accompanied by variable probabilities of footshock punishment (Simon et al., 2011). These findings imply that flupenthixol, through blocking the D1 receptor type, could have reduced risky choice, predominantly in risk-prone rats characterized by higher levels of D1 mRNA expression. Our data provide indirect support to this notion. In this experiment, there was a subgroup of rats showing risk-prone behavior under vehicle treatment (≥50% risky lever preference in the 12.5% trial block) in which flupenthixol almost significantly reduced the preference for the risky lever (*p* = 0.06). Thus, this latter finding leaves open the possibility of a contribution of AcbS DA activity in mediating risk-based decision making, possibly through D1 receptor signaling in risk-prone rats as suggested by data from Simon et al. (2011).

### Orbitofrontal Dopamine and Risky Choice

Intra-OFC flupenthixol did not alter risk-based decision making or win-stay/lose-shift tendencies, an observation that might not be accounted for by inappropriate drug dosing. For instance, similar or lower doses of flupenthixol infused into the adjacent prelimbic cortex were behaviorally effective in related tasks (Naneix et al., 2009). However, we cannot rule out that higher doses could impair risky choices. Of note, previous studies using the same task suggest that it is the medial (Stopper et al., 2014a), not the most lateral part of the OFC (St Onge and Floresco, 2010), that mediates risk-based decision making. In our study, microinfusions were aimed at more ventral regions of the OFC, shown to mediate behavioral flexibility in instrumental tasks through DA D1-, D2-, and N-methyl-D-aspartate-receptor-mediated signaling (Bohn et al., 2003; Calaminus and Hauber, 2008). In view of the drug spread observed after prefrontal microinfusion of the radio-labeled DA D1 receptor antagonist SCH23390 in the same volume (0.5 µl) as used here (Granon et al., 2000), our flupenthixol microinfusions should have targeted not only lateral and ventral but also medial OFC regions, though at somewhat more posterior locations, as in the study by Stopper et al. (2014a). A recent study revealed that, by increasing reward sensitivity and behavioral flexibility, OFC DA depletion improved the ability of marmosets to learn a visual discrimination task that involved ambiguous feedback (Clarke et al., 2014). Furthermore, there is correlative evidence for a role of OFC DA signaling in modulating decision making, since greater D2 mRNA expression in the OFC predicts higher risk preference (Simon et al., 2011). However, our findings provide no evidence for a role of OFC DA activity in risk-related decision making.

### Brain Dopamine and Risky Choice

The main finding of our study is that intra-AcbC D1/D2 activity plays a critical role in promoting choice of the risky reward. While it is clear that D1/D2 receptor-mediated signals must support risky choice by influencing the activity of AcbC neurons, the nature of this influence is difficult to assess. DA neurons operate in distinct temporal modes: they display responses across varying timescales, including fast phasic changes of DA release on a time scale of seconds and slower phasic changes in a range of minutes, while tonic levels of DA provide a DA receptor tone (for review, see Schultz, 2007; Hauber, 2010). Intra-AcbC flupenthixol likely disrupted phasic as well as tonic DA signaling, thus a blockade of one or more of these informational and modulatory DA signals could underlie the shift in risky choices seen here. For instance, a subset of DA neurons display slow and moderate activation that increases gradually during the interval between a reward-predicting stimulus and reward delivery and varies monotonically with risk (Fiorillo et al., 2003). However, due to its low magnitude, this DA risk signal probably induces only a relatively low DA release that might predominantly activate D2 receptors that are mostly in a high affinity state, but not the low affinity D1 receptors (Schultz, 2010). Given that intra-AcbC D1 receptor-mediated signaling may be particularly relevant to support the risky choices analyzed here, a contribution of this DA risk signal to promote risk seeking seems therefore less likely. A recent study in rats performing a risky choice task revealed that phasic DA signals provide feedback on whether prior actions were rewarded, signals that are critical to update risk-related decision-making policies (Stopper et al., 2014b). As intra-AcbC flupenthixol might have blocked the phasic DA signals that provide feedback both on risky reward and certain reward actions, it is difficult to see how such a drug effect would produce the observed preference for the certain lever. Of note, our rats subjected to AcbC flupenthixol were able to track increasing risks associated with the large reward but, importantly, displayed a generally reduced preference for the risky lever across all trial blocks, including the initial trial block (4 pellets at *p* = 1.0). This suggests that AcbC flupenthixol produced a general bias away from the lever known to provide an initially certain large reward but that becomes increasingly risky within a session. In other words, intra-AcbC flupenthixol could have rendered animals more sensitive to the known costs of the risky response option. Remarkably, AcbC flupenthixol failed to mimic the effects of systemic flupenthixol: rats that received systemic flupenthixol displayed a high preference for the risky lever in the initial trial block but not later trial blocks, suggesting that they may be more sensitive to changes in risk.

Thus, AcbC activity in intact rats could provide information about the overall cost-benefit ratios of the available response options in more general terms. In line with this notion, recent microdialysis studies revealed that slow fluctuations of tonic Acb DA in the range of minutes incorporate multiple types of information, such as reward availability and risk (Stopper et al., 2013). However, it is important to note that the flupenthixol-induced shift in risky choices could not only reflect a blockade of such informational DA signals but a blockade of modulating DA signals as well. Tonic DA activity provides an enabling influence (Schultz, 2007) and modulates excitatory input from the mPFC, OFC, and BLA, regions that are critical in mediating risk-based decision making (Mobini et al., 2002; Ghods-Sharifi et al., 2009; St Onge and Floresco, 2010). Accordingly, the flupenthixol-induced bias toward the safe response option could also reflect a dysregulated integration of afferent information from cortical and limbic regions in the AcbC. Moreover, the mPFC, BLA, and OFC each receive prominent input from midbrain DA neurons that subserve action selection. For instance, DA signals in the BLA support reward-directed action (Berglind et al., 2006): however, their role in risk-based decision making is still unknown. By contrast, DA signals in the mPFC govern risky choice by complementary actions on D1 and D2 receptors (St Onge et al., 2011). Here we show that DA signals in the OFC, though supporting flexible reward-directed responding (e.g. Calaminus and Hauber, 2008; Takahashi et al., 2009; Clarke et al., 2014), may not modulate risky choices. Thus, the mPFC and OFC are essential components of a neural circuit mediating risk-based decisions; however, processes in the OFC that subserve risky decisions do not rely on intact DA input, other than those in the mPFC.

## Conclusions

Here we show that, among the terminal areas of DA projections investigated, DA activity in the AcbC plays a critical role in promoting the choice of the risky reward. The observed pattern of responding suggests that intra-AcbC DA D1/D2 receptor signaling does not support the ability to track increasing risk associated with the large reward but invigorates responding and inclines rats to choose a response option known to be associated with increasing risk-related costs. This conclusion is consistent with the largely congruent concepts that AcbC DA activity invigorates reward seeking (Nicola, 2007), mediates a “go” response toward motivationally-relevant stimuli (Floresco, 2015), or serves as a bridge that enables animals to traverse the distance that separates them from reward in multiple dimensions, including risk or effort (Salamone and Correa, 2012). In addition, our data provide support to the idea that drug actions in the AcbC play a major role in mediating abnormal risk-related decision making in humans under DA agonist therapy (Cools et al., 2003; Gallagher et al., 2007).

## Supplementary Material

For supplementary material accompanying this paper, visit http://www.ijnp.oxfordjournals.org/

## Statement of Interest

The authors declare no potential conflict of interest.
